# Perceptions, facilitators and barriers of digital interdisciplinary consultation: a qualitative study

**DOI:** 10.1093/fampra/cmaf074

**Published:** 2025-09-29

**Authors:** C Y Hidding, P Buist, K Peeters, J Cals, J Jansen, N D Scherpbier-de Haan, J Stoffelen, M H Blanker, H van der Worp, S M Sanavro, S M Grol, H J Schers

**Affiliations:** Department of Primary and Community Care, Radboud Institute for Health Sciences, Radboud University Medical Centre, P.O. Box 9101, Nijmegen 6500 HB, The Netherlands; Department of Primary and Long-Term Care, University Medical Centre Groningen, P.O. Box 196, Groningen 9700 AD, The Netherlands; Department of Family Medicine, Care and Public Health Research Institute, Maastricht University, Maastricht 6229 ER, The Netherlands; Department of Family Medicine, Care and Public Health Research Institute, Maastricht University, Maastricht 6229 ER, The Netherlands; Department of Family Medicine, Care and Public Health Research Institute, Maastricht University, Maastricht 6229 ER, The Netherlands; Department of Primary and Long-Term Care, University Medical Centre Groningen, P.O. Box 196, Groningen 9700 AD, The Netherlands; Zorgbelang Inclusief, Arnhem 6811 JH, The Netherlands; Department of Primary and Long-Term Care, University Medical Centre Groningen, P.O. Box 196, Groningen 9700 AD, The Netherlands; Department of Primary and Long-Term Care, University Medical Centre Groningen, P.O. Box 196, Groningen 9700 AD, The Netherlands; Department of Primary and Long-Term Care, University Medical Centre Groningen, P.O. Box 196, Groningen 9700 AD, The Netherlands; Department of Primary and Community Care, Radboud Institute for Health Sciences, Radboud University Medical Centre, P.O. Box 9101, Nijmegen 6500 HB, The Netherlands; Department of Primary and Community Care, Radboud Institute for Health Sciences, Radboud University Medical Centre, P.O. Box 9101, Nijmegen 6500 HB, The Netherlands

**Keywords:** access to care, multidisciplinary care, consultation, doctor–patient relationship, primary care

## Abstract

**Background:**

The demand for care is increasing in volume and complexity. Digital interdisciplinary consultation (DIDC) has the potential to support general practitioners (GPs) with specialist knowledge and prevent unnecessary referrals. Successful DIDC requires collaboration between stakeholders with potentially different goals, barriers and motivations. Little is known about this broader context of DIDC.

**Aim:**

To identify stakeholders' perceptions about DIDC including barriers and facilitators to its use.

**Design and setting:**

Semistructured interviews with seven stakeholder groups in the Netherlands.

**Method:**

We used purposive sampling to attain various stakeholders. The semistructured online or telephone interviews were transcribed verbatim and analyzed thematically.

**Results:**

We interviewed 46 stakeholders (12 GPs, 10 medical specialists, 11 patients, 3 health insurance representatives, 6 platform developers, 3 primary–secondary care coordinators (PSCCs), and 1 chief medical information officer). We identified six themes: effectiveness, implementation requirements, evaluation of DIDC use, impact on networked care, acceptance by patients and future use of DIDC. Overall, stakeholders were positive about DIDC. The expected impact of DIDC on the accessibility and affordability of healthcare varied from modest to high. Agreements on collaboration and funding, and the need for user-friendly digital platforms were identified as the most important requirements for successful implementation. Physicians particularly appreciated the asynchronous nature of DIDC and the time saved. Patients appreciated receiving timely care, free from a deductible excess and avoiding unnecessary hospital visits.

**Conclusion:**

This study shows that all stakeholders were overall positive about DIDC. Agreements on organization, collaboration, financing, and platform design are essential.

Key messages

- Stakeholders generally appreciated digital interdisciplinary consultation (DIDC).
- Collaboration and funding agreements are important requirements.
- User-friendliness is essential for sustained and effective platform use.
- Physicians particularly appreciate the asynchronous nature.
- Despite limited involvement, DIDC is highly appreciated by patients.

## Introduction

In the Netherlands, the demand for care is increasing in volume and complexity, which, without changes, will reach the limits of financial, human, and social sustainability in the future [1, 2].

Digital interdisciplinary consultation (DIDC) is defined as asynchronous, targeted communication between healthcare providers via a secure electronic application where patient-specific information is shared and clarification or guidance is sought with regard to a clinical care question. The intended effects of this easily accessible specialist knowledge are to strengthen the role of primary care providers by increasing their knowledge, to provide more accessible care [3–5] and to reduce the number of referrals and make them more accurate [4, 6, 7]. DIDC has potential benefits for patients such as time and cost savings [4, 7] and it can guide patients to the right place for the right care, contributing to accessible and affordable healthcare [3, 8].

Since the introduction of teledermatology in the Netherlands in 1995, the use of DIDC has increased [9]. Several types of digital platforms have been developed, ranging from a one-on-one consultation, mostly used in a regional setting, to a national networking platform, called Prisma, where anonymous casuistry can be discussed multidisciplinarily [7, 10]. Nine different digital consultation platforms are currently available in the Netherlands. Despite this increase in use and available platforms, little is known about the usability, benefits and drawbacks of DIDC. Although the literature suggests an overall positive attitude [7], health care professionals, patients, health care insurers and platform developers, may have different goals, motivations, and barriers. Gaining insight into the drivers for the use of DIDC, and how they vary between stakeholders is important [11, 12].

In this interview study, we explored stakeholders' views, perceived barriers, facilitators and values of DIDC for its acceptance, implementation and use.

## Materials and Methods

### Design and setting

We conducted an interview study, including stakeholders for DIDC. We used a qualitative thematic analysis based on a phenomenology research approach. The Standards for Reporting Qualitative Research framework [13] and the Consolidated Criteria for Reporting Qualitative Health Research (COREQ, Supplement S1) [14] were used for an explicit and comprehensive reporting of this study.

Data collection took place through semistructured interviews. Literature findings and the research question shaped the dynamic interview guides (Supplement S2). Developed by the research team and refined in three pilot interviews, the interview guides focused on identifying barriers and facilitators and exploring visions on DIDC among stakeholders in the Netherlands.

### Population and recruitment

General practitioners (GPs), medical specialists, health insurance representatives, patients, developers of digital consultation platforms, primary–secondary care coordinators (PSCCs) and chief medical information officers (CMIOs) were selected via purposeful sampling. A CMIO is a physician with knowledge of ICT who connects these two domains. We approached potential participants via the research team's professional network, through snowball sampling and via calls on LinkedIn and the national networking platform Prisma [7, 10]. Details on participant recruitment can be found in Table 1.

**Table 1.** Methods of recruitment per stakeholder group.

Stakeholder	Methods of recruitment
General practitioners	Professional network, AHON (UMC Groningen), CEL (Maastricht University), AHN (Radboudumc)
Medical specialists	Professional network, Prisma, LinkedIn, snowball sampling
Platform designers	Contacting relevant organization
Health insurance companies	Contacting health insurance companies
Patients	Zorgbelang Inclusief, LinkedIn, via participating GPs
CMIO	Snowball sampling
Primary–secondary care coordinators (PSCC)	Professional network, snowball sampling

We excluded online discussion forums without questions about specific patients, general email communication unless explicitly described as secure, video conferencing, one-way communication, and services that connect patients and caregivers [7]. Heterogeneity was sought in terms of age, gender, geography, type of employment, viewpoints on DIDC, and use of digital communication platforms. The use of DIDC by GPs and medical specialists was mandatory. We aimed for an equitable distribution of (ex-)users and nonusers of the *Prisma* network platform as this platform is distinguished by its structure and approach. Participants received a compensation of €100 for their participation.

### Data collection

Between 1 August 2022 and 10 March 2023, the first author, GP and junior researcher, conducted all interviews after three pilot interviews. With consent, audio recordings were made of the interviews that took place via Microsoft Teams or telephone. Field notes were taken during and after the interviews. The audio recordings were transcribed verbatim and participants were given the option to proof-read the pseudonymized transcripts.

The number of interviews per group was preferentially determined by achieving data saturation [15].

### Data analysis

A thematic analysis was conducted using Atlas-ti (versions 22.0.11–24.0.0) [16, 17] through two rounds of independent coding by three coders. The coders were supervised by a senior researcher experienced in qualitative research and coding. Details on the coders and supervisor can be found in Supplement 1 (COREQ). After the initial round, consensus on coding language was achieved. In the second round, this coding scheme was established after coding one interview per stakeholder in duplicate. Latent, *in vivo* and process codes were used.

After coding 11 interviews, we created and refined themes in our research group until consensus was reached.

## Results

### Characteristics of the participants

Characteristics of the 46 interviewees are presented in Table 2. Fifty percent of the GPs and specialists were current or former users of the Prisma network platform, while the other half had no experience with it.

**Table 2.** Characteristics of the interviewed stakeholders.

Stakeholder	GPs	Medical specialists	Health insurance companies	Patient (representatives)	Platform developers	CMIO	Primary–secondary care coordinators (PSCC)
Number of participants	*n* = 12	*n* = 10	*n* = 3	*n* = 11	*n* = 6	*n* = 1	*n* = 3
Age (Average in years (range))	47 (35–67)	49 (40–61)	47 (37–62)	63 (32–88)	46 (39–58)	34 (N/A)	54 (45–59)
Women/men	7/5	3/2	1/2	6/5	1/5	0/1	2/1
Experience as a doctor (Average in years (range))	16 (2–35)	14 (7–25)	N/A	N/A	N/A	5 (N/A)	N/A
(ex-)user of network platform Prisma (n (% (ex-) users))	6 (50%)	5 (50%)	N/A	N/A	N/A	1 (100%)	N/A
Level of education (n (highly educated%))	12 (100%)	10 (100%)	3 (100%)	5 (45%)	6 (100%)	1 (100%)	3 (100%)

### Key themes

Six themes were found: effectiveness, implementation requirements, evaluation of DIDC use, impact on networked care, acceptance by patients and future use of DIDC.

#### Theme 1 effectiveness

The importance of effective healthcare organization was stressed by all stakeholders. Positive contribution of DIDC to the future accessibility and affordability of healthcare through fewer, more accurate referrals, was expected.

*“It (DIDC) contributes to the affordability of care […]. Although sometimes it is difficult to correlate the two, so the moment you look at a reduction in costs, […], we do see a decrease in the participating practices, but it is sometimes difficult to draw the conclusion that it is 100% linked.”*

*- Health insurer, Z03*

All stakeholders expected growth of GP expertise and a decreasing future need for DIDC, as a result of a learning effect and refresher courses based on frequently asked questions. Many mentioned DIDC's contribution to the timeliness of care through rapid specialist responses.

“*… substitution of care means for GPs: we get more work. […] and we don't get paid for it. […] If you contrast the substitution of care with the picture of increasingly expensive care that cannot be afforded by the public, it suits us that we organise care efficiently and that we give care in an echelon where it is easily possible. This also means that substitution is a positive thing.”*

*- GP, B08*

On the other hand, GPs mentioned that DIDC is not a part of their routine yet and takes time. Medical specialists often made commitments on response speed and incorporated DIDC into their working day. Nonetheless, some medical specialists were afraid of missing questions or delaying urgent medical cases.

#### Theme 2: implementation requirements

Table 3 provides an overview of the requirements for a successful implementation of DIDC per stakeholder.

**Table 3.** Overview of most important requirements for DIDC implementation per stakeholder.

Category of requirement	Stakeholder					
	GP	Medical specialist	PSCC	Platform developer	Patient	Health insurer
**Financial requirements**	Appropriate remuneration for users of DIDC	Appropriate remuneration for users of DIDC	Appropriate remuneration for users of DIDC	Appropriate remuneration for users of DIDC		
	No deductible excess health insurance for patients			No deductible excess health insurance for patients	No deductible excess health insurance for patients is a plus, no strict requirement	No deductible excess health insurance for patients
	Leaving separate financing of primary and secondary health care for networked care	Leaving separate financing of primary and secondary health care for networked care	National agreements on funding for DIDC	National agreements on funding for DIDC		Finding a good funding structure for primary–secondary care projects/networked care
	No subscription costs for the use of digital platforms		Agreements on DIDC funding	Financial support from health insurers		DIDC should not increase healthcare expenses
**Software requirements**	Great ease of use	Great ease of use	Great ease of use	Great ease of use		
	Link with GPs medical program, no complex login procedure	Notifications for new DIDC in agenda or otherwise	Link with GPs medical program	Link with GPs medical program		New DIDC initiatives should be well researched
		Patient anonymity in multidisciplinary consultation			Patient anonymity in multidisciplinary consultation	
	Ensuring data security	Ensuring data security	(not discussed in interview)	Ensuring data security	Ensuring data security	(not discussed in interview)
	Limited time investment of approx. 10' per DIDC	Limited time investment of approx. 10' per DIDC	Limited time investment of approx. 10' per DIDC			
**Organizational requirements**	Hospital-wide implementation of a digital platform	Hospital-wide implementation of a digital platform	Hospital-wide implementation of a digital platform	Hospital-wide implementation of a digital platform		
	Clarity on medical responsibility and liability	Clarity on medical responsibility and liability		Clarity on medical responsibility and liability		
	National collaboration for complex casuistry	National collaboration for complex casuistry		National collaboration for complex casuistry	Multidisciplinary digital consultation for complex casuistry	National collaboration for complex casuistry
	Regional collaboration is preferred, especially when referring after DIDC-use	Regional collaboration important	Regional collaboration important	Regional collaboration important, especially when referring after DIDC-use		Support regional collaboration with existing good primary–secondary care, in clearly defined areas
	Providing time to use DIDC	Providing time to use DIDC				
**Collaboration agreements**	Suitable advice for use in primary care setting	On subsequent referral, enclose digital advice	On subsequent referral, enclose digital advice			Increasing job satisfaction for physicians
	Response time	Response time	Response time	Response time		
	Type of casuistry	Type of casuistry	Type of casuistry		Type of casuistry	

##### Financial requirements

A form of remuneration for physicians using DIDC was generally considered appropriate, although some physicians and patients questioned the need for it. GPs desired no subscription costs for the use of digital platforms. Much was still unclear about the effects on healthcare costs.

“*Of course, there are fixed criteria (which DIDC should meet). In principle, it shouldn't make quality worse, it shouldn't make care much more expensive, and accessibility is also important, that it eventually gets better. […] Job satisfaction is also really the fourth factor that is very important to us.”*

*- Health insurer, Z01*

PSCCs and physicians involved in the implementation process, considered the lack of structural, national financing agreements for DIDC as one of the main barriers. Health insurance companies acknowledged this and were challenged to find a financial translation for networked care.

One of the success factors and requirements for DIDC use is that it does not affect the patient's deductible excess. Although beneficial, this was not a requirement for patients.

“*The deductible excess would not stop me from not seeing a medical specialist, because I think my health is just too important for that. […] That's not where it would stop me.”*

*- Patient, P02*

##### Software and ICT requirements

When asked about, the security of medical data was confirmed by physicians and platform developers to be a requirement. While all stakeholders seemed aware of the potential risks, there appeared to be little concerns about data security and privacy. Platform developers indicated that this was protected by legislation and regulations in the Netherlands.

“*I think that if there are people who find a disadvantage to it (digital versus telephone consultation), is that they might have a fear of data being out on the street. […] I take it that the majority think it's okay.”*

*- Patient, P13*

The lack of standardization of medical data for exchange and the fragmentation of the ICT landscape were main barriers for platform developers. Solutions for standardization were provided [18].

“*… when designing technology for healthcare providers, on the one hand you have to ensure that unambiguity is created so that you can store that data in a very structured way […], but you want the (technological) support in daily practice to have a certain degree of freedom…”*

*- Platform developer, B03*

Platform developers did not mention any bottlenecks for platform development, as theoretically, almost anything is technically possible.

Complex access procedures were one of the main barriers. GPs and, to a lesser extent, medical specialists expected easy access. In addition, ease of use was one of the main requirements for physicians to use DIDC.

“*… if your waiting room is full […], you are not interested if the login process takes more than a minute.”*

*- GPs’ viewpoint according to PSCCs, B02*

##### Organizational requirements

GPs, platform developers and PSCCs considered hospital-wide implementation important, so different specialties could be consulted within one digital platform.

Most GPs, unlike medical specialists, expected to be able to use DIDC within working hours. Most medical specialists did not mind answering DIDC-questions after hours, mainly because of the low volume of questions.

##### Collaboration agreements

Most stakeholders stressed the importance of clear agreements on the use of DIDC addressing finances, platform, type of casuistry, content of Q&A, and response time.

“*I believe that when you bring them (GPs and specialists) together, […] you learn to understand each other a little bit better and when you understand each other, you get an exponential increase in potential gains.”*

*- platform developer, B15*

“*What helps a lot with this kind of communication is that hospitals have a hospital-wide policy and GPs also have a GP-wide policy […], you make joint agreements and as a GP you have to be a reliable partner and so does the hospital.”*

*- GP, B08*

Clarity on medical responsibility and liability, and guaranteeing the quality of care were also considered important prerequisites. All GPs felt that the medical responsibility after use of DIDC remained with them. Most felt strongly supported in making clinical decisions by the specialists' advice. To some medical specialists, it was unclear where their responsibilities lied and this was, together with the fear of missing out on (urgent) cases, a reason to be reluctant to use DIDC. Making well-defined agreements about the type of casuistry and the time span in which an answer can be expected is important. With patient safety in mind, it is important not to discuss urgent casuistry, which seemed clear to GPs, but was essential to medical specialists to use DIDC confidently. PSCCs endorsed clarity on the aforementioned topics as a prerequisite for successful DIDC implementation.

“*If you refer a patient, then you (GP) are the lead physician until the patient is seen for the first time by that specialist. This is a bit of a grey area at DIDC. In particular, the patient has to know very clearly where to go if something is wrong, […].”*

*- platform developer, B15*

#### Theme 3: evaluation of DIDC use

DIDC was most often used to coordinate or avoid a referral, to obtain advice and to minimize telephone contact. For both GPs and medical specialists, avoiding interfering telephone calls for nonurgent questions was one of the main advantages of DIDC. Additionally, being able to reach part-time colleagues efficiently through DIDC was an advantage.

The interviewed physicians were satisfied with the ease of use and the time spent formulating questions or answers. Medical specialists noted the importance of a good notification-system. The agreed response time, usually two to three working days, deemed appropriate. Physicians were satisfied with the type of questions and usability of the answers.

Some platforms' chat functions enabled specialists to ask follow-up questions, but most physicians disliked lengthy digital conversations.

Physicians and PSCCs expected better quality of care via DIDC compared to consulting a medical specialist by telephone. GPs indicated they could present a case more completely with DIDC, although some experienced difficulties adding nuances to a written case.

“*They asked several questions, including: “what do you think of the quality?” And they (GPs) did indeed say that the advice is better substantiated. It's also nice that I get it in writing and that I can include the advice in my file.”*

*- PSCC, B09*

Although DIDC did not appear to reduce the perceived workload, both GPs and specialists experienced increased job satisfaction.

“*A bit of a contradiction perhaps, but I think the job satisfaction has increased because, on the one hand, it makes the work more diverse and […] you get a better feeling for what is going on in primary care. […]On the other hand […] I think the pressure of work has only increased because of it […], even more places where I can be questioned, even at times that do not suit me at all.”*

*- Medical specialist, S11*

#### Theme 4: impact on networked care

DIDC contributed to a better primary–secondary care collaboration according to physicians, PSCCs and platform developers. Most physicians prioritized contributing to better regional collaboration over better national collaboration, except for advice on rare pathologies and for subspecializations.

“*What we have now concurred that we in that [name platform] group say: I agree with my colleague. That turns out to have added value for GPs: I asked, one cardiologist thinks so, someone else from another part of the country agrees. […] So I think that absolutely makes a difference for patients as well.”*

*- Medical specialist, S09*

#### Theme 5: acceptance by patients

Patients were not directly involved in the process of DIDC, making it an abstract concept for them to give their opinion on. Most patients appreciated it when their GP sought additional advice from a medical specialist. How this information was sought was less important. As a result, there was a high level of acceptance for the use of DIDC, also among nondigitally literate patients.

“*Whether you look up literature yourself or consult a specialist digitally, the fact that you want to put effort into treating the patient as best as possible is greatly appreciated.”*

*- GP, H12*

“*As a patient, I don't think it even matters that much how a healthcare provider gets his information. […] I do think I like to hear as a patient “I have consulted with colleagues” […]. For me, that's more confidence-building. […] I can also imagine that some patients don't find it confidence-building at all, because they think: oh, he (GP) doesn’t know.”*

*- Patient, P07*

Patients highly valued the time- and cost savings of avoiding a hospital visit. Patients were not required to take time off work, saved on travel and healthcare costs, and were satisfied with the timeliness of care.

“*It (DIDC) saves me a visit to the specialist. It enriches the knowledge and the arguments on which the GP makes his decisions. […] I think that's actually a very good development.”*

*-Patient, P07*

Although some patients valued a physical appointment with a medical specialist, most GPs felt that patients, particularly older-, palliative-, and frail patients, were better served by medical care close to home. Most patients interviewed agreed with this.

“*We see increasing multimorbidity among vulnerable people. […] If one generalist GP can provide that care and, if that doesn't work, can easily consult a specialist, then that patient is better off.”*

*- GP, B08*

Acceptance of DIDC was mainly determined by the relationship of patients with their GP. Even when they were worried, most patients found the use of DIDC acceptable.

“*To make healthcare more efficient, […] better coordination, so also to get more patient centered care and that a medical specialist doesn't have to be involved in everything […], that can work fine.”*

*- Patient, P09*

Patients generally did not expect to be involved in the DIDC application and relied on the expertise and feedback of GPs.

#### Theme 6: the future of DIDC

Most stakeholders believed that DIDC will become a permanent feature of the healthcare landscape. There was no perceived need to move toward a single, national platform, as all platforms had their strengths and weaknesses. It was suggested that DIDC could be made mandatory in the future, prior to a referral. In that case, the medical specialist could convert a DIDC into a referral, or a referral could be handled with digital advice.

## Discussion

### Summary

Stakeholders agreed on DIDC's potential to improve healthcare accessibility, affordability, and timely care. Clear agreements on medical responsibility and finance, secure data handling, and ease of use are critical for successful implementation. DIDC enhances GP-specialist collaboration and job satisfaction, even in the absence of workload reduction. Patients appreciated DIDC for saving time and costs, valuing the GP relationship. It is expected that in the future DIDC will become a permanent part of healthcare delivery, potentially streamlining referrals and consultations.

### Strengths and limitations

The focus in this study was on GPs, medical specialists, and patients. We aimed for data saturation in all stakeholder groups, but this was not achieved for health insurance representatives, platform developers, PSCCs and CMIOs. The first two groups were limited by the number of people available or involved in the subject, which led to population saturation. Due to limited resources, we did not expand on the other two stakeholder groups. As a result, we may lack further insights into the IT aspects, practical considerations related to the implementation of DIDC and potential primary–secondary care collaboration-hiccups.

When selecting the participants, we searched for diverse views on DIDC to question proponents and opponents of the concept. In retrospect, critics toward the subject might be underrepresented. Especially regarding the opinions of GPs and medical specialists this has some limitations, although we found that also the more critical subjects were not completely negative or strongly opposed against the idea of DIDC. For patients, DIDC is an abstract concept because they are not involved in the process. Therefore, it was not easy to find patients willing to be interviewed about DIDC. Through the help of GPs, Zorgbelang Inclusief and LinkedIn, we were able to interview 11 patients on the subject, which gave us new insights.

### Comparison with existing literature

The results of this study show a generally positive attitude toward DIDC across all stakeholder groups. This is in accordance with literature findings [4, 6, 7]. Previous studies from abroad and on a smaller scale in the Netherlands appear to have drawn up a positive cost-benefit balance [8, 19, 20]. The interviews with the stakeholders only partially covered cost-effectiveness and economic evaluation. However, it is well-known that for most stakeholders these are important factors for wide-scale acceptance and implementation. Nonetheless, to date limited is known about the true cost savings of DIDC [4, 6, 21]. This was confirmed in the interviews with health insurers, PSCCs and platform developers, even though a positive effect was expected. However, also for health insurers, cost savings were not the main goal of DIDC. The various stakeholders were looking for an effective and pleasant collaboration tool that increases job satisfaction and contributes to appropriate patient care. DIDC is associated with improved access to hospital care and a reduction in referrals, although this conclusion may not be unequivocally drawn based on current scientific evidence [22].

Physicians have varying opinions about the influence on workload. On the one hand, DIDC can relieve workload due to easy access of the platforms, the asynchronous aspect and time savings compared to telephone contact. Additionally, DIDC may lead to fewer referrals to specialists. On the other hand, specialists expressed a fear of being overwhelmed by the number of questions and GPs fear the increase of patient care due to the avoided referrals. We also find these varying experiences and expectations in literature [23–25].

Little can be found in (inter)national literature on the views and experiences of medical specialists, but they seem to have limited enthusiasm [4, 7]. A recent interview study revealed more enthusiasm, in line with our findings [25].

GPs and medical specialists both stressed the importance of clarity on medical responsibility in the DIDC process. All GPs in our study felt responsible. Most specialists felt partially responsible, but often did not know exactly what responsibility they bore. The importance of clarity about this is supported in other studies [4, 9, 26].

Benefits to the patient include timely specialist advice, reduced costs, and elimination of travel and parking expenses. Patient satisfaction therefore appears to be high, as was found in the literature [4, 5].

### Implications for practice

It is important that collaboration agreements are established and that financial, organizational and platform requirements are met for the successful implementation of DIDC. For those involved in DIDC implementation, the main barrier is the lack of national agreements on DIDC funding. The ease of use of DIDC may paradoxically act as a barrier, possibly generating more questions than necessary. In addition, there is currently insufficient evidence on the cost savings resulting from the use of DIDC.

## Conclusion

The user-friendliness of currently available platforms, the time commitment when using DIDC and the timeliness of providing care satisfies physicians and patients. In addition, the avoidance of phone calls between physicians, due to the asynchronous nature of DIDC, contributes significantly to overall satisfaction. Although the effects of DIDC on affordability and accessibility of care are often perceived as limited, stakeholders are positive about the use and potential of DIDC. For successful implementation, it is advisable to establish robust collaboration agreements that cover financial aspects, type of caseload with particular attention to patient safety, response times, and other relevant factors.

Although we have identified strengths and limitations of DIDC and mapped the most beneficial way for it to be used according to different stakeholders, further research is needed into the effect on healthcare costs, accessibility, effectivity and quality of care. Additionally, an analysis of which casuistry is suitable, is desired. This research contains indicators of the factors that could ensure a successful development and implementation of DIDC. Further investigation of which specific aspects of DIDC are important for future evaluation and valuation is advised.

## Supplementary Material

cmaf074_Supplementary_Data

## Data Availability

The data underlying this article will be shared on reasonable request to the corresponding author.
