# Supplementary material for: Perceptions, facilitators and barriers of digital interdisciplinary consultation: a qualitative study
Source: Fam Pract. 2025 Sep 29;42(5):cmaf074. doi: 10.1093/fampra/cmaf074 (PMC12478473; doi:10.1093/fampra/cmaf074)
Supplement: cmaf074_Supplementary_Data [file cmaf074_supplementary_data.zip › Supplement 2 interview guide health insurance.pdf]

Interview guide health insurance company

Prior to the start of the interview:

- Thank you for participating in this study and for making time for this interview.
- Introducing interviewer and organisation of the study.
- The purpose of this study is to identify how various stakeholders, including yourself as a representative of one of the Dutch health insurers, see the use of digital interdisciplinary consultation between GPs and medical specialists.
- An audio recording of the interview will be made. This recording and data will be processed confidentially and anonymously. After transcription of the interview, you can read back your answers if you wish and correct any ambiguities or answers meant differently.
- Please feel free to answer the questions asked openly and honestly, there are no right or wrong answers and greatly appreciate your willingness to cooperate with our research.
- After starting the recording, I will not mention your name but I will mention your interview number. In total, this interview will take 45 - 60 minutes.
- **With your permission, I will now start the audio recording.**

- **After starting recording, please mention interview number.**

Introduction

Age: Gender: M/F
Representing which health insurance:

In general, how do you feel about the increasing degree of digitisation within healthcare?

1. Attitude towards digital interdisciplinary consultation.

What do you think about the possibility of consultation between GP and medical specialist being done digitally? Why?

Are there any applications whose implementation is supported by name health insurance? Why?

Do you know of any other digital consultation applications?
→ Why supported/not supported by [name health insurance]?

Is the quality of care delivered in this way assessed in some way? How?

Do you have insight into the impact of this way of working on care?
Don't be too quick to make suggestions but think, for example:

- influence on (the number of) referrals
- speed with which patients are helped
- impact on waiting lists
- delay or faster diagnosis of potentially serious conditions
- etc.

2. Promoting and restraining factors for putting digital interdisciplinary consultation into practice.

Are new ways of digital peer-to-peer consultation currently under development?

Are these or other future projects supported by name health insurance? In what way? Why or why not?

When deciding whether or not to support certain ways of digital consultation, what is all taken into account?

What do you and/or name health insurance expect from a digital consultation platform?

Are there certain criteria imposed when developing such digital applications?
(For example, think about quality, safety of care provided and data retention).

How is this tested?

3. Suggested outcome measures for future assessment of these (relatively) new forms of interdisciplinary consultation.

What do you expect for the future of healthcare with regard to digitisation?
(Do you see it as a necessity or rather a blessing?)

In your opinion, what would be good outcome measures to measure the value of a digital consultation platform or application in the future?

We covered all the topics I wanted to discuss with you. Are there any questions or comments you would like to share?

Thank you again for your participation.

Stop recording.
