# Supplementary material for: Perceptions, facilitators and barriers of digital interdisciplinary consultation: a qualitative study
Source: Fam Pract. 2025 Sep 29;42(5):cmaf074. doi: 10.1093/fampra/cmaf074 (PMC12478473; doi:10.1093/fampra/cmaf074)
Supplement: cmaf074_Supplementary_Data [file cmaf074_supplementary_data.zip › Supplement 2 interview guide medical specialist.pdf]

Interview guide medical specialist

Prior to the start of the interview:

- Thank you for participating in this study and for making time for this interview.
- Introducing interviewer and organisation of the study.
- The purpose of this study is to identify how various stakeholders, including yourself as a medical specialist, see the use of digital interdisciplinary consultation between GPs and medical specialists.
- An audio recording of the interview will be made. This recording and data will be processed confidentially and anonymously. After transcription of the interview, you can read back your answers if you wish and correct any ambiguities or answers meant differently.
- Please feel free to answer the questions asked openly and honestly, there are no right or wrong answers and greatly appreciate your willingness to cooperate with our research.
- After starting the recording, I will not mention your name but I will mention your interview number. In total, this interview will take 45 - 60 minutes.
- **With your permission, I will now start the audio recording.**

- **After starting recording, please mention interview number.**

Introduction

Age: Gender: M/F

Specialism: Number of years active:

Type of centre: UMC - small regional hospital - larger regional hospital

Prisma user: yes/no?

In general, how do you feel about an increasing degree of digitalisation within healthcare and the ability to consult digitally with your colleagues within primary care?

Are there any applications you are aware of and do you use? Why?

How extensively do you use these digital consultations?

1. Attitude towards digital interdisciplinary consultation.

How do you feel about being able to consult or answer GPs' questions in this way?

How have you experienced the use of digital consultation?

Do you have confidence in this way of working?

Do you also get to know the patient's questions and/or preferences when dealing with a digital consultation question?

Does this way of working affect collaboration with GPs in the region? In what way?

Are medical responsibilities clear to you?

What do you think about this?

What do you think about the quality of care provided with this way of working?

How do you experience the ease of use and quality of the digital applications you use?

Do you find that digital interdisciplinary consultation affects workload?

If so, in what way?

What do you do when the supplied cases and/or questions are unclear and/or incomplete?

2. Promoting and restraining factors for putting digital interdisciplinary consultation into practice.

What elements play a role in using these platforms more or less?

In terms of content, what do you expect from a digital consultation platform?

What would be a motivator or incentive for you to have interdisciplinary consultation in this way?

What would be a barrier or obstacle for you to use a digital platform?

3. Suggested outcome measures for future assessment of these (relatively) new forms of interdisciplinary consultation.

What do you expect for the future of healthcare with regard to digitisation?
(Do you see it as a necessary tool or rather as a blessing?)

Do you plan to continue using digital consultation in the future? To what extent?

According to you, what would be good outcome measures/parameters to measure the value of a digital consultation platform or application in the future?

We covered all the topics I wanted to discuss with you. Are there any questions or comments you would like to share?

Thank you again for your participation.

Stop recording.
