# Supplementary material for: Perceptions, facilitators and barriers of digital interdisciplinary consultation: a qualitative study
Source: Fam Pract. 2025 Sep 29;42(5):cmaf074. doi: 10.1093/fampra/cmaf074 (PMC12478473; doi:10.1093/fampra/cmaf074)
Supplement: cmaf074_Supplementary_Data [file cmaf074_supplementary_data.zip › Supplement 2 interview guide patient(representatives).pdf]

Interview guide patient (representative)

Prior to the start of the interview:

- Thank you for participating in this study and for making time for this interview.
- Introducing interviewer and organisation of the study.
- The purpose of this study is to identify how various stakeholders, including yourself as a patient(representative), see the use of digital interdisciplinary consultation between GPs and medical specialists.
- An audio recording of the interview will be made. This recording and data will be processed confidentially and anonymously. After transcription of the interview, you can read back your answers if you wish and correct any ambiguities or answers meant differently.
- Please feel free to answer the questions asked openly and honestly, there are no right or wrong answers and greatly appreciate your willingness to cooperate with our research.
- After starting the recording, I will not mention your name but I will mention your interview number. In total, this interview will take 45 - 60 minutes.
- **With your permission, I will now start the audio recording.**

- **After starting recording, please mention interview number.**

Introduction

Age: Gender: M/F

Highest level of education:

Profession:

Region:

In general, how do you feel about the digitalisation within healthcare?

1. Attitude towards digital interdisciplinary consultation.

How is the relationship with your GP?

How do you feel about your GP seeking advice from a medical specialist?

Are you aware that your GP can also consult a medical specialist digitally?

What do you think of this possibility?

There is also a possibility to submit medical casuistry anonymously to a large group of medical specialists and other GPs via a medical digital platform called Prisma. How do you feel about consultations taking place in this way?

How do you think this will or could affect the quality of care?

How do you think this would affect the position/image of GPs?

With an increasing demand for care and scarcity of GPs, there may be a greater role for digital care in the future. How do you feel about this?

2. Promoting and restraining factors for putting digital interdisciplinary consultation into practice.

What would be reasons for you to allow digital consultation between your GP and a medical specialist? Why?

And what reasons would stand in your way? Why?

3. Suggested outcome measures for future assessment of these (relatively) new forms of interdisciplinary consultation.

What do you expect for the future of healthcare with regard to digitisation?
(Do you see it as a necessity or rather a blessing?)

In your opinion, what would be good ways to measure the value of a digital consultation between GP and specialist in the future?

What is important to you when delivering care via digital consultation between GP and specialist? What aspects do you value highly?

(Do not give suggestions too quickly but think for example about speed with which you were helped, whether or not you like to continue to be treated by GP, safety of care, privacy,...)

We covered all the topics I wanted to discuss with you. Are there any questions or comments you would like to share?

Thank you again for your participation.

Stop recording.
