# Supplementary material for: Perceptions, facilitators and barriers of digital interdisciplinary consultation: a qualitative study
Source: Fam Pract. 2025 Sep 29;42(5):cmaf074. doi: 10.1093/fampra/cmaf074 (PMC12478473; doi:10.1093/fampra/cmaf074)
Supplement: cmaf074_Supplementary_Data [file cmaf074_supplementary_data.zip › Supplement 2 interview guide platform developer.pdf]

Interview guide platform developer

Prior to the start of the interview:

- Thank you for participating in this study and for making time for this interview.
- Introducing interviewer and organisation of the study.
- The purpose of this study is to identify how various stakeholders, including yourself as a developer of digital consultation platforms, see the use of digital interdisciplinary consultation between GPs and medical specialists.
- An audio recording of the interview will be made. This recording and data will be processed confidentially and anonymously. After transcription of the interview, you can read back your answers if you wish and correct any ambiguities or answers meant differently.
- Please feel free to answer the questions asked openly and honestly, there are no right or wrong answers and greatly appreciate your willingness to cooperate with our research.
- After starting the recording, I will not mention your name but I will mention your interview number. In total, this interview will take 45 - 60 minutes.
- **With your permission, I will now start the audio recording.**

- **After starting recording, please mention interview number.**

Introduction

Age: Gender: M/F

Which digital platform are you involved/working at?

How long have you been involved?
Were you involved from the start of the development or did you join the company later?

Can you briefly describe the possibilities for GPs when using [name application]?

In general, how do you feel about the increasing degree of digitisation within healthcare?

1. Attitude towards digital interdisciplinary consultation.

What do you think about the possibility of consultation between GP and medical specialist being done digitally? Why?

You are involved in the development to enable this way of consultation. What drives you in this regard?

Do you have insight into the impact this way of working will have on healthcare?
(Don't be too quick to make suggestions but think, for example: influence on (the number of) referrals, speed with which patients are helped, influence on waiting lists, etc.)

2. Promoting and restraining factors for putting digital interdisciplinary consultation into practice.

Are new ways of digital peer-to-peer consultation currently under development?

- New teleconsultation within existing platform?
- Other means of consultation?
- What are the advantages and disadvantages of these?

What are factors you consider when developing digital consultation platforms?

Are you familiar with facilitating factors that encourage use by doctors?
Do you know why this is experienced that way?

Do you take this knowledge into account when developing or adapting your application?

Are you familiar with the barriers experienced by users of digital platforms?
Do you know why this is experienced that way?

Do you take this knowledge into account when developing or adapting your application?

Are there certain criteria that are imposed when developing such digital applications?
(For example, think about quality, safety of care delivered and data retention).
How is this tested?

3. Suggested outcome measures for future assessment of these (relatively) new forms of interdisciplinary consultation.

What do you expect for the future of healthcare with regard to digitisation?

Where do you see missed opportunities?

Where do you see potential or opportunities when it comes to digital communication between healthcare providers?

In your opinion, what would be good outcome measures to measure the value of a digital consultation platform or application in the future?

We covered all the topics I wanted to discuss with you. Are there any questions or comments you would like to share?

Thank you again for your participation.

Stop recording.
