# Supplementary material for: Perceptions, facilitators and barriers of digital interdisciplinary consultation: a qualitative study
Source: Fam Pract. 2025 Sep 29;42(5):cmaf074. doi: 10.1093/fampra/cmaf074 (PMC12478473; doi:10.1093/fampra/cmaf074)
Supplement: cmaf074_Supplementary_Data [file cmaf074_supplementary_data.zip › Supplement 1 COREQ 32.pdf]

COREQ (COnsolidated criteria for REporting Qualitative research) Checklist

Domain 1: research team and reflexivity						
Personal characteristics						
Initials of coders/interviewer			C.Y.H.	P.B.<br>coder	L.A.M.<br>coder	S.M.G.<br>coder and supervisor
1	Interviewer/facilitator	Which author/s conducted the interview or focus group?	C.Y.H.			
2	Credentials	What were the researcher's credentials? *E.g. PhD, MD*	MSc.	MSc.	MSc.	PhD., MSc.
3	Occupation	What was their occupation at the time of the study?	Junior researcher and GP	Junior researcher	Junior researcher	Senior researcher, health scientist, policy consultant
4	Gender		Female	Female	Female	Female
5	Experience and training	What experience or training did the researcher have?	Qualitative Research Methods & Analysis course (E. Rap)	n.a.	Introduction of qualitative research by Jeanine Evers	Senior experience and training in qualitative research methods
Relationship with participants						
6	Relationship established	Was a relationship established prior to study commencement?	Yes			
7	Participant knowledge of the interviewer	What did the participants know about the researcher?	Prior to the interview, an introductory meeting took place in which the professional background of the interviewer and the purpose of the study were discussed, among other things.			
8	Interviewer characteristics	What characteristics were reported about the interviewer?	Professional background, experience as a GP and as a researcher.			
Domain 2: Study design						
Theoretical framework						
9	Methodological orientation and theory	What methodological orientation was stated to underpin the study?	Phenomenology			
Participant selection						
10	Sampling	How were participants selected?	Purposive and snowball sampling			
11	Method of approach	How were participants approached?	e-mail, Linked-in, through professional network			
12	Sample size		46			

13	Non-participation	How many people refused to participate or dropped out?	Few people were refused because data-saturation had been reached.
Setting			
14	Setting of data collection	Where was the data collected?	Online interviews via Microsoft Teams where possible and if not possible (e.g. with older people) a telephone interview was held
15	Presence of non-participants	Was anyone else present besides the participants and researchers?	No
16	Description of sample	What are the important characteristics of the sample?	Heterogeneity was sought in terms of age, gender, geography, type of employment and use of digital communication platforms. The use of DIDC by GPs and medical specialists was mandatory.
Data collection			
17	Interview guide	Were questions, prompts, guides provided by the authors? Was it pilot tested?	Different interview guides were used for the different stakeholders. Three pilot interviews were done prior to the interviews.
18	Repeat interviews	Were repeat inter views carried out? If yes, how many?	No.
19	Audio-visual recordings	Did the research use audio or visual recording to collect the data?	Audio-recordings were made.
20	Field notes	Were field notes made during and/or after the interview?	During and immediately after the interviews, field notes were taken by the interviewer.
21	Duration	What was the duration of the interviews?	Approx. 45 to 90 minutes per interview.
22	Data saturation	Was data saturation discussed?	Yes.
23	Transcripts returned	Were transcripts returned to participants for comment and/or correction?	Yes, although there was no obligation to proofread and/or comment on the transcript, all participants were given this opportunity.
Domain 3: analysis and findings			
Data analysis			
24	Number of data coders	How many data coders coded the data?	4
25	Description of the coding tree	Did authors provide a description of the coding tree?	A coding tree was not predefined but derived from the data.
26	Derivation of themes	Were themes identified in advance or derived from the data?	Derived from the data.
27	Software	What software, if applicable, was used to manage the data?	Atlas-ti (versions 22.0.11- 24.0.0)

28	Participant checking	Did participants provide feedback on the findings?	No, only on the transcripts of their own interviews.
Reporting			
29	Quotations presented	Were participant quotations presented to illustrate the themes/findings? Was each quotation identified?	Yes, several illustrative quotes from all stakeholder groups were displayed in the article with personal coding, anonymous but unique to each participant.
30	Data and findings consistent	Was there consistency between the data presented and the findings?	Yes.
31	Clarity of major themes	Were major themes clearly presented in the findings?	Yes.
32	Clarity of minor themes	Is there a description of diverse cases or discussion of minor themes?	Yes.

Developed from: Tong A, Sainsbury P, Craig J. Consolidated criteria for reporting qualitative research (COREQ): a 32-item checklist

for interviews and focus groups. International Journal for Quality in Health Care. 2007. Volume 19, Number 6: pp. 349 – 357
