# Supplementary material for: Perceptions, facilitators and barriers of digital interdisciplinary consultation: a qualitative study
Source: Fam Pract. 2025 Sep 29;42(5):cmaf074. doi: 10.1093/fampra/cmaf074 (PMC12478473; doi:10.1093/fampra/cmaf074)
Supplement: cmaf074_Supplementary_Data [file cmaf074_supplementary_data.zip › Supplement 2 interview guide GP.pdf]

Interview guide general practitioner

Prior to the start of the interview:

- Thank you for participating in this study and for making time for this interview.
- Introducing interviewer and organisation of the study.
- The purpose of this study is to identify how various stakeholders, including yourself as a GP, see the use of digital interdisciplinary consultation between GPs and medical specialists.
- An audio recording of the interview will be made. This recording and data will be processed confidentially and anonymously. After transcription of the interview, you can read back your answers if you wish and correct any ambiguities or answers meant differently.
- Please feel free to answer the questions asked openly and honestly, there are no right or wrong answers and greatly appreciate your willingness to cooperate with our research.
- After starting the recording, I will not mention your name but I will mention your interview number. In total, this interview will take 45 - 60 minutes.
- **With your permission, I will now start the audio recording.**

- **After starting recording, please mention interview number.**

Introduction

Age:	Number of years active as a GP:
Gender: M/F	Type of employment:
Type of practice:	number of GPs
	other staff

Location (rural/urban/type of patients (think education level, age, type of neighbourhood/village))

Prisma user: yes/no

In general, how do you feel about the increasing degree of digitisation within healthcare and the possibility of digital consultation with your colleagues within the hospital?

Are there any applications you are aware of and do you use? Why yes/no.
How extensively do you use digital interdisciplinary consultation?

1. Attitude towards digital interdisciplinary consultation.

How do you feel about being able to consult with the hospital in this way?

How have you experienced the use of digital interdisciplinary consultation?

Does this form of consultation also affect collaboration with colleagues in the hospital? In what way?

How are the answers/data obtained recorded in your patients' medical file?
How do you feel about this?

Are the medical responsibilities clear to you?
What do you think about this?

What do you think about the quality of care provided with this way of working?
(Think of impact on continuity of care, person-centred, safe, effective, efficiency, timely response and accessibility, etc.)

How do you experience the ease of use and quality of the digital applications you use?

Do you find that digital interdisciplinary consultation affects workload? In what way?

In your opinion, does this form of communication affect the image of the GP?

Does it affect your relationship with your patient? If yes, in what way?

How do you deal with answers that are unclear or out of line?

Is there a learning effect for yourself? How do you value this?

In what way is the patient involved in the decision to request a digital consultation?

In what way do you communicate the advice obtained to the patient?

How do you follow up on outstanding questions?
(Have you built in a safety net? Does the follow-up lie with yourself, the patient, the assistant, etc.?)

2. Promoting and restraining factors for putting digital interdisciplinary consultation into practice.

What elements play a role in using these platforms more or less?

In terms of content, what do you expect from a digital consultation platform?
(Consider ease of use, link with medical file, specialist response time, privacy concerns, among others).

What would be a motivator or incentive for you to have the interdisciplinary consultation in this way?

What would be a barrier or obstacle for you to use a digital platform?

3. Suggested outcome measures for future assessment of these (relatively) new forms of interdisciplinary consultation.

What do you expect for the future of healthcare with regard to digitisation?
(Do you see it as a necessity or rather a blessing?)

Do you plan to continue using digital consultation in the future?
To what extent?

In your opinion, what would be good outcome measures/parameters to measure the value of a digital consultation platform or application in the future?

We covered all the topics I wanted to discuss with you. Are there any questions or comments you would like to share?

Thank you again for your participation.

Stop recording.
